# Synthesis and Characterization of a (−)-Epicatechin and Barbituric Acid Cocrystal: Single-Crystal X-ray Diffraction and Vibrational Spectroscopic Studies

**DOI:** 10.1021/acsomega.0c06239

**Published:** 2021-03-16

**Authors:** Iwona Budziak-Wieczorek, Urszula Maciołek

**Affiliations:** † Department of Chemistry, University of Life Sciences in Lublin, Akademicka 15, 20-950 Lublin, Poland; ‡ Analytical Laboratory, Institute of Chemical Sciences, Faculty of Chemistry, Maria Curie-Sklodowska University, pl. M. Curie-Skłodowskiej 3, 20-031 Lublin, Poland

## Abstract

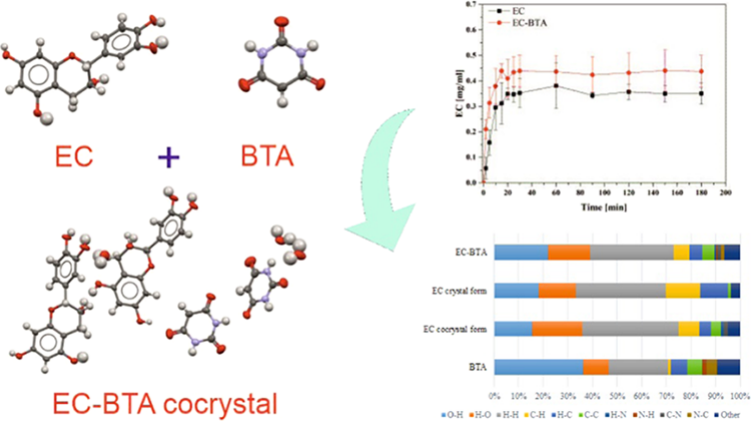

The paper presents the contribution of the cocrystallization method in the physicochemical modification of catechins that exhibit low oral bioavailability. This was done to obtain cocrystals for two naturally occurring polyphenolic diastereoisomers (+)-catechin and (−)-epicatechin with commonly used coformers. Due to distinct crystallization behavior, only the (−)-epicatechin cocrystal with barbituric acid in a 1:1 stoichiometry was obtained. The cocrystal of (−)-epicatechin (EC) with barbituric acid (BTA) was prepared by the slow solvent-evaporation technique. The structure and intermolecular interactions were determined by X-ray crystallographic techniques. The analysis of packing and interactions in the crystal lattice revealed that molecules in the target cocrystal were packed into tapes, formed by the O–H···O type contacts between the (−)-epicatechin and coformer molecules. The EC molecules interact with the carboxyl group in the BTA coformer mainly by −OH groups from the benzene ring A. The cocrystalline phase constituents were also investigated in terms of Hirshfeld surfaces. The application of Raman spectroscopy confirmed the involvement of the C=O group in the formation of hydrogen bonds between the (−)-epicatechin and barbituric acid molecules. Additionally, the solubility studies of pure EC and the EC-BTA cocrystal exhibited minor enhancement of EC solubility in the buffer solution, and pH measurements confirmed a stable level of solubility for EC and its cocrystal.

## 1 Introduction

A number of methods are known to improve the pharmacokinetic profile of bioactive compounds, for example, using salts, amorphous dispersions in a polymer matrix, an inorganic matrix as a carrier, nanoparticles, and cocrystals.^1^ Especially, crystal engineering and cocrystallization techniques appear as a promising way to modify the bioavailability (e.g., solubility, permeability, and stability) and other physicochemical properties for a wide range of bioactive compounds.^2−6^ The main component of a cocrystal is the active pharmaceutical ingredient (API), which is associated with the coformer through noncovalent interactions, such as hydrogen bonds, electrostatic interactions, halogen bonds, π–π stacking, and van der Waals forces.^7−9^ Another important factor is the stability of bioactive compounds, and, in this case, the cocrystals should be thermodynamically much more stable than the pure API forms.^10^ Recently, crystal engineering has played an important role in the context of modifying the bioavailability of natural compounds like flavonoids.

Polyphenols, which are secondary metabolites of plants, are found largely in fruits, vegetables, cereals, and beverages.^11−14^ Owing to their antioxidant properties, physiological activities, and health-promoting effects on the human body, they have attracted a growing interest in the human diet.^13,15^ However, despite promising health applications, their bioavailability has been limited due to poor adsorption into the bloodstream and the need for sufficiently high plasma concentrations to elicit their favorable health effects.^15^ Because solubility and bioavailability are fundamentally important properties in drug discovery, formulation, and crystallization, searching for new methods to increase the limited bioavailability of flavanoids has generated a lot of interest recently.

One of the promising groups of polyphenol compounds are catechins, which are present in large amounts in beverages such as red wine, tea, and cocoa-based products and are characterized by a wide spectrum of health-promoting properties. Additionally, polyphenolic catechins exhibit a relatively low oral bioavailability; only 0.2–2% of their intake amount reaches the bloodstream.^16^ The maximum catechin plasma concentration of tea catechins is obtained about 2 h after consumption and is up to 1–2 μmol/L.^17^ Several factors can influence the low bioavailability of catechins including potential sensitivity to digestive conditions, poor intestinal transport, rapid metabolism, and bioconversions.^17,18^

**Figure 1:**
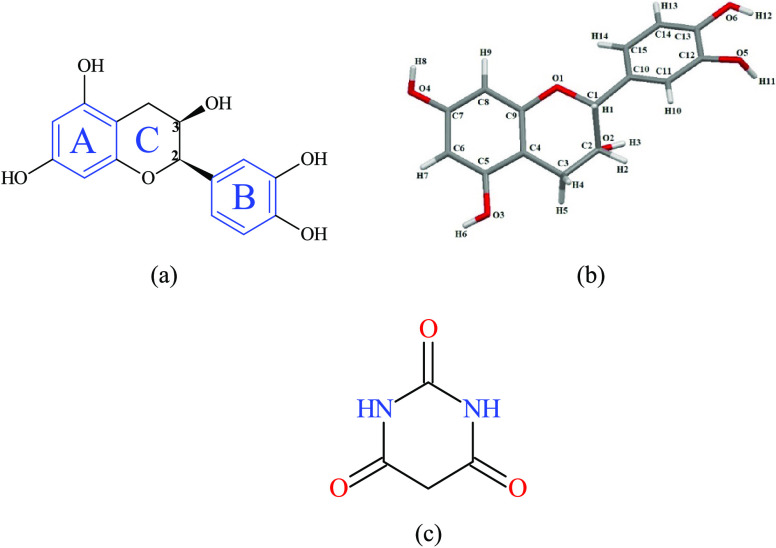
Scheme of the (−)-epicatechin (EC) structure (panel a), atom numbering in the EC cocrystal used in this paper (panel b), and the scheme of coformer barbituric acid (BTA) (panel c).

An example of such a compound is (−)-epicatechin (EC), which has confirmed health-promoting properties, including antioxidant, anti-inflammatory, antibacterial, anticarcinogenic, antihypertensive, neuroprotective, and cholesterol-lowering ones.^19−24^ In its structure, (−)-epicatechin possesses two benzene rings called A and B and a dihydropyran ring (C ring) with a hydroxyl group on the C3 carbon, as well as two chiral centers on the C2 and C3 carbons (Figure 1).^25^ EC is highly soluble in water, but membrane permeability is reported to be low.^26^ Thus, novel methods for improving the oral bioavailability of catechins are desirable. So far there have been no reports on any cocrystal form of EC in the Cambridge Structural Database (CSD), besides the crystal structure of pure EC.

The main goal of this study was to prepare cocrystals of two natural diastereoisomers (+)-catechin and (−)-epicatechin with commonly used coformers (barbituric acid, 5-methylo-1*H*-benzotriazole, 4-(methyloamino)-pyridine, 5-methylbenzimidazole, acetamide, salicylamide, niacinamide, isonicotinamide, caffeine, and glutarimide) and investigate the molecular structures and intermolecular interactions using single-crystal X-ray diffractions. These two molecules possess different conformations, which could affect cocrystallization behavior. Finally, one cocrystal of EC with barbituric acid (BTA) was successfully obtained through the slow evaporation of the solvent. Additionally, barbituric acid and particularly its derivatives, commonly known as barbiturates, have been a topic of interest due to their significance in biology, medicine, and supramolecular chemistry.^27,28^ Although barbituric acid itself has no pharmaceutical applications, barbiturates exhibit anesthetic as well as hypnotic properties and are used as antidepressants for the central nervous system.^29,30^ BTA can be used as a coformer in cocrystallization studies due to structural simplicity and donor–acceptor features related to the presence of the amide group (Figure 1).^3^

The resulting cocrystal was characterized by single-crystal X-ray diffraction, powder X-ray diffraction, differential scanning calorimetry, and Raman spectroscopy. Moreover, the dissolution behavior in PBS buffer and different pH environments was investigated to compare the behavior of the cocrystal and the pure component.

## 2 Results and Discussion

### 2.1 Single-Crystal X-ray Diffraction

The synthesis of cocrystals was performed for the two naturally occurring polyphenolic diastereoisomers (+)-catechin and (−)-epicatechin with 10 different coformers. However, these compounds display structural similarities, as well as significantly distinct crystallization behavior.^31^ In this case, a good-quality cocrystal was obtained only with (−)-epicatechin with the barbituric acid coformer. Further in the paper, only the results for EC-BTA are shown.

#### 2.1.1 EC Molecule in the Cocrystal

**Figure 2:**
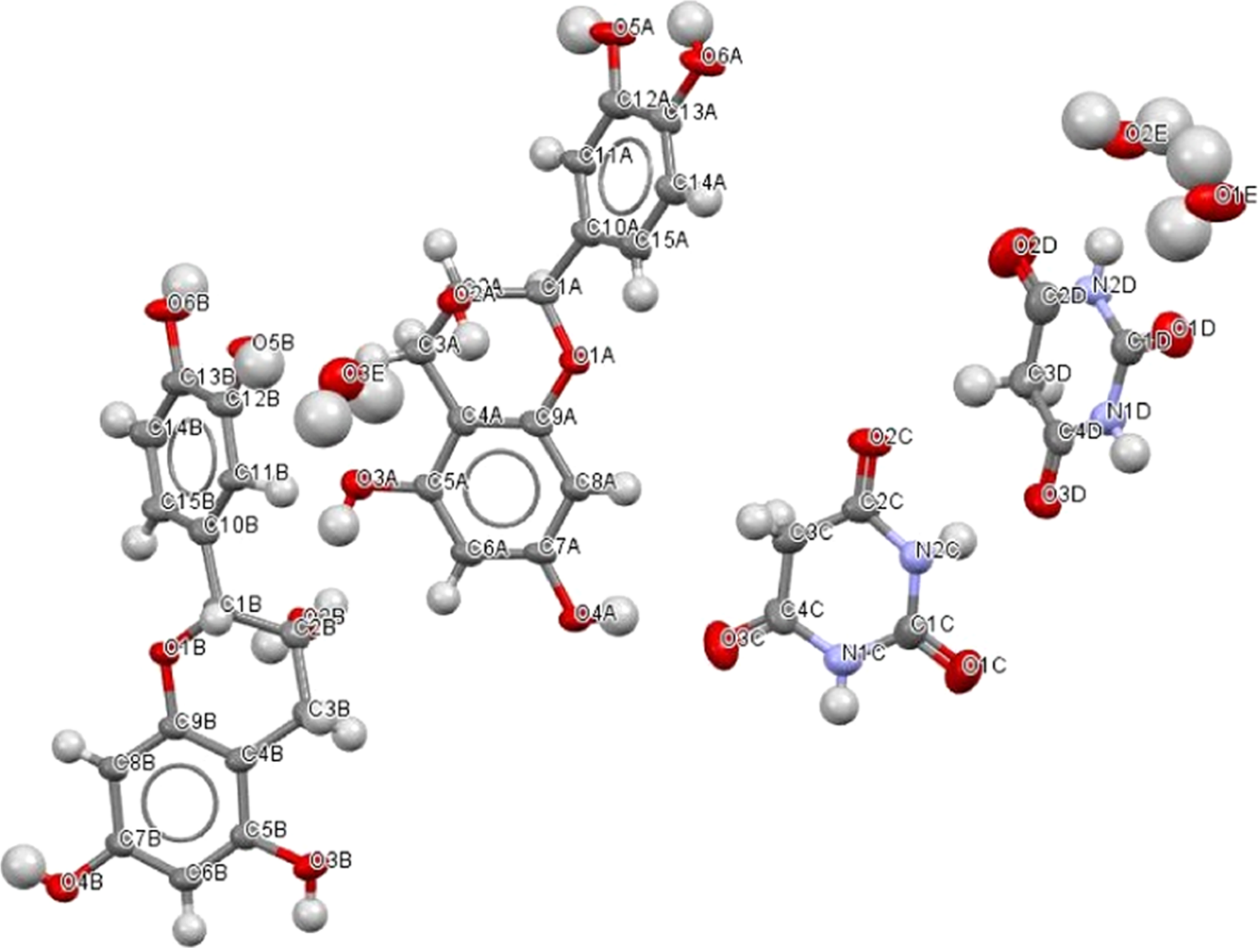
Asymmetric unit of EC-BTA, showing the nonhydrogen atom-numbering scheme. Displacement ellipsoids are drawn at 50% probability level.

The crystal structure was determined using single-crystal X-ray diffraction. The obtained data revealed that the cocrystal EC-BTA crystallizes in the triclinic space group *P*1 with two molecules each of EC and BTA and three water molecules in the asymmetric unit, as shown in Figure 2. The data related to the cell parameters and the final structural refinement are shown in Table 1.

**Table 1:** Crystallographic Parameters and Refinement Details for the (−)-Epicatechin Cocrystal

compound	EC-BTA (1:1)
empirical formula	C_38_H_42_N_4_O_21_
temperature (K)	293(2)
crystal system	triclinic
space group	*P*1
*a* (Å)	7.3043(5)
*b* (Å)	11.8154(8)
*c* (Å)	12.0064(6)
α (deg)	67.444(6)
β (deg)	87.953(5)
γ (deg)	88.503(5)
*V* (Å^3^)	956.23(11)
*Z*	1
calculated density (g cm^3^)	1.547
absorption coefficient (mm^–1^)	1.100
*F*(000)	467.9
2θ range for data collection (deg)	7.98–147.66
index ranges	–9 ≤ *h* ≤ 8
	–14 ≤ *k* ≤ 11
	–14 ≤ *l* ≤ 10
reflections collected	6303/4382 (*R*_int_ = 0.0518)
data/restraints/parameter	4387/3/596
goodness of fit on *F*^2^	1.080
final *R* indices *R* [*I* > 2σ(*I*)]	*R*_1_ = 0.0593
	*wR*_2_ = 0.1554
final *R* indices (all data)	*R*_1_ = 0.0670
	*wR*_2_ = 0.1779
largest diff. peak and hole/e Å^–3^	0.17/–0.27
CCDC number	2047274

The bond lengths and dihedral angles for the EC molecules are very similar to those of other catechins (see Table 2).^32^ The (−)-epicatechin molecule has a nonplanar conformation given by the dihedral angles about O1C1C10C15 (−20.6°) and O1C1C10C11 (162.9°). This is caused by participation of the −OH group in the intermolecular hydrogen bond formation. The angle between the planes in the rings A and B in the structure of the EC molecule is 37.04°. The O1–C1 (1.438 Å) and O1–C9 (1.375 Å) bonds in ring C are asymmetrical due to the conjugation effect on the C_9_ side. The aromatic C–C bond lengths are typically in the range from 1.38 to 1.41 Å. The bond length list for all molecules is presented in the Supporting Information (Table S1).

**Table 2:** Bond Distances and Dihedral Angles for the EC Molecules (Molecule A: Red)

atoms	length/Å	geometric parameters	dihedral angles (deg)
O1–C1	1.438(6)	O1–C1–C10–C15	–20.6(5)
O1–C9	1.375(6)	O1–C1–C10–C11	162.9(4)
O2–C2	1.412(6)		
O3–C5	1.358(6)		
O4–C7	1.362(6)		
O5–C12	1.357(7)		
O6–C13	1.372(6)		
C1–C2	1.520(8)		
C2–C3	1.528(6)		
C3–C4	1.492(7)		
C4–C5	1.411(7)		
C5–C6	1.396(7)		
C6–C7	1.382(7)		
C7–C8	1.385(7)		
C8–C9	1.388(7)		
C4–C9	1.393(7)		
C1–C10	1.516(6)		
C10–C11	1.379(7)		
C11–C12	1.383(7)		
C12–C13	1.409(7)		
C13–C14	1.366(8)		
C14–C15	1.380(7)		
C15–C10	1.390(7)		

**Figure 3:**
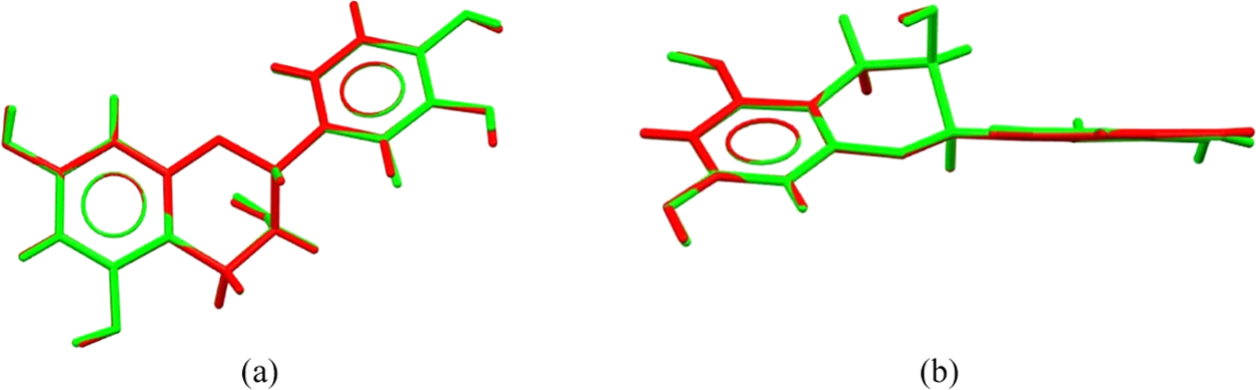
Superposition of two EC molecules in a direct way. Side and top views of the EC molecules in EC-BTA (panels a and b).

In the crystal lattice of the EC-BTA cocrystal, there are two symmetry-independent EC molecules, A (in red) and B (in green) (Figure 3). The superposition of these molecules shows an almost perfect overlap, with only slight differences in the arrangements of the hydrogen atoms from those of hydroxyl groups. This may be due to differences in the strength of the hydrogen bonds formed.

#### 2.1.2 Crystal Lattice Description

**Figure 4:**
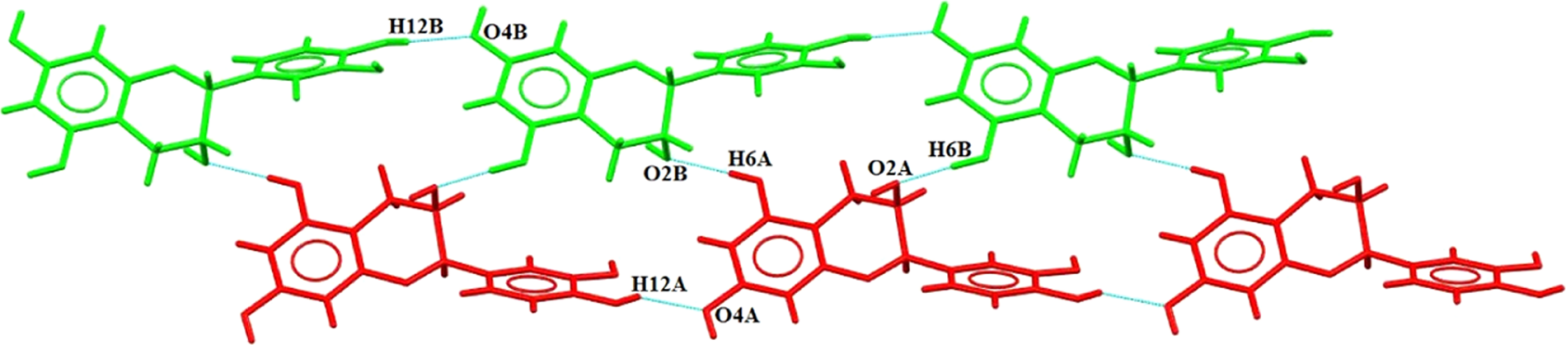
Layers of two symmetry-independent EC molecules in the EC-BTA cocrystal.

In the crystal lattice of the EC-BTA cocrystal, two symmetry-independent EC molecules form a ribbon through hydrogen bond O–H···O networks along the layers and between them (Figure 4). The system is stabilized by intermolecular hydrogen bonds among atoms O6A–H12A···O4A, O6B–H12B···O4B, O3B–H6B···O2A, and O3A–H6A···O2B. The strongest hydrogen bonds occur between molecules A (red) and B (green), O3B–H6B···O2A (1.784 Å) and O3A–H6A···O2B (1.935 Å), and they are responsible for the EC layers’ stabilization.^33^

#### 2.1.3 Packing and Intermolecular Interactions

**Figure 5:**
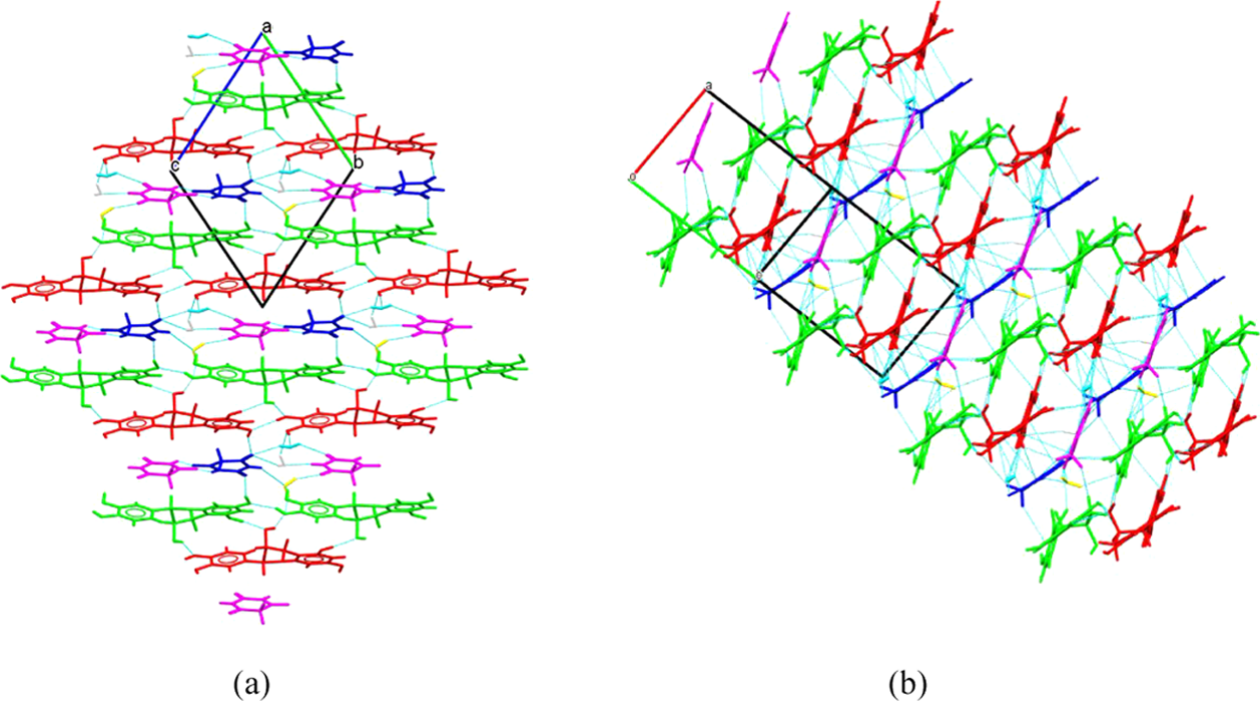
Crystal packing and interactions in the EC-BTA cocrystal: panel a, the view along the [100] direction and panel b, the view along direction [01̅1].

**Figure 6:**
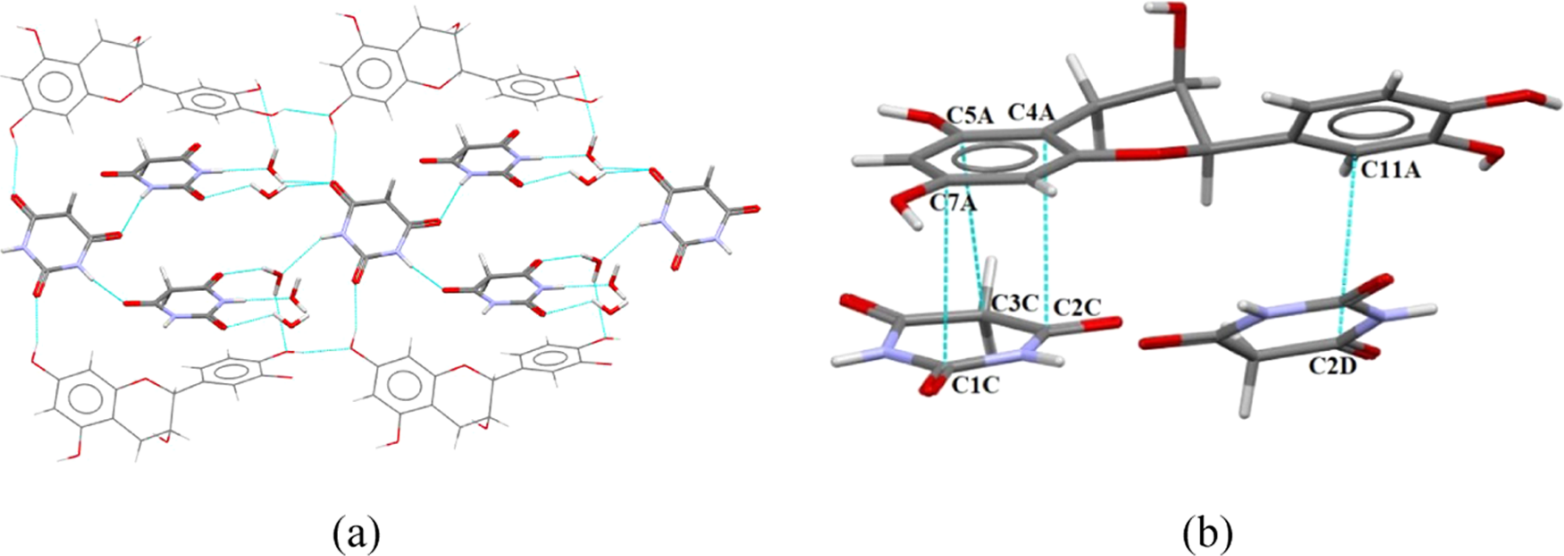
Hydrogen bonds between EC and BTA molecules (panel a) and stacking interactions in the EC-BTA cocrystal (panel b).

The packing of molecules and their weak interaction motifs in the crystal structure are presented in Figure 5 in panels a and b. The crystal structure of EC-BTA is composed of EC molecule ribbons separated by BTA and water molecules having the same almost planar orientation. The EC molecules interact with the BTA molecules mainly through the O–H···O hydrogen bonds (Figure 6a). The hydrogen atom H8 from the OH group from benzene ring A in the EC molecule forms a hydrogen bond with the carboxyl group C=O from barbituric acid: O4B–H8B···O1C and O4A–H8A···O3C. The BTA molecules interact with each other weakly (short contact) and through hydrogen bonds O···H–N and N–H···O. Additionally, the BTA molecules interact indirectly through water molecules associated with the cocrystal structure. Moreover, in the crystal lattice, weak π···π interactions between the EC molecules (red species) and the barbituric acid ring (distance in the range of 3.362–3.387 Å) can be observed. This type of interaction is presented in Figure 6b.

### 2.2 Powder X-ray Diffraction Analysis

The PXRD patterns of the EC-BTA cocrystal, the physical mixture, and pure (−)-epicatechin and barbituric acid are shown in Figure S1 in the Supporting Information. EC-BTA displayed unique crystalline peaks when compared to the EC and the BTA coformer. Formation of the cocrystalline phase leads to the appearance of numerous diffraction peaks at 11.8, 13.4, 13.0, 14.7, 16.1, 16.9, and 22.0°, which are absent in the reflection patterns of EC and BTA, and the peaks at 15.1, 18.0, and 20.3° in BTA shifted toward 15.3, 18.2, and 20.5°, respectively, in the EC-BTA cocrystal. Sets of reflections 19.4 and 19.7°, 23.6 and 24.2°, and 26.0 and 26.3° merged in the EC-BTA cocrystal. In this case, the presence of distinguishable reflections in the PXRD pattern confirms the formation of a new cocrystalline phase of EC-BTA.

### 2.3 Thermal Analysis

Thermal properties of the EC-BTA cocrystal were assessed by means of the melting point and TGA–DSC analysis in relation to the individual components to investigate stability and phase transitions.

The melting point of an API can be modified by forming cocrystals, and in most cases, cocrystals reveal melting points between those of the API and the coformer or lower than that of the API or the coformer.^10,34,35^ The melting points of pure (−)-epicatechin, barbituric acid, and cocrystal were determined and were 240.5, 254, and 227 °C, respectively. The melting point of EC-BTA was lesser than those of its individual components. The reduced melting point of the cocrystal is due to changes in molecular interactions between (−)-epicatechin and barbituric acid, different packing arrangements, and changes in crystallinity in comparison to those of the individual components.^36^

The TGA–DSC plots for the pure component and the EC-BTA cocrystal are presented in the Supporting Information in Figure S2. (−)-Epicatechin and barbituric acid showed a single endothermic peak at 245.04 and 252.64 °C, respectively, which is consistent with the reported melting point. On the DSC curve of the EC-BTA cocrystal, the first endothermic peak at around 121.34 °C is attributed to the water/solvent loss. The other endothermic peak at 214.95 °C indicates the melting point of the cocrystal, which is significantly different from those of (−)-epicatechin and the coformer. This distinct thermal behavior with different melting transitions between the cocrystal and the individual components confirms the formation of a new solid phase.

### 2.4 Raman Spectroscopy

**Figure 7:**
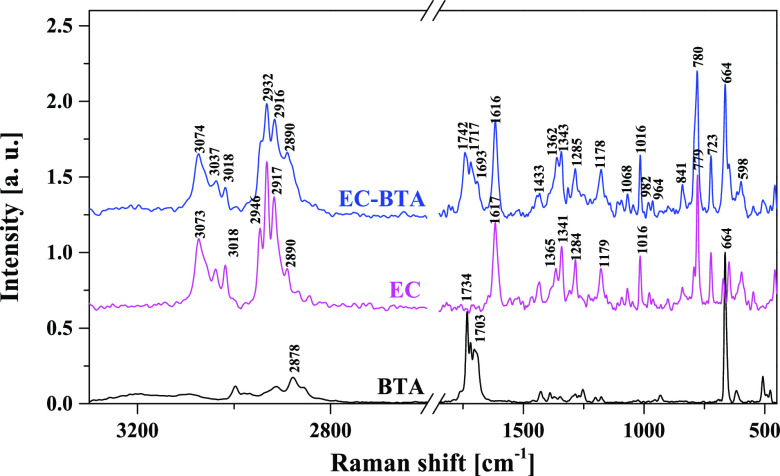
Raman spectra of (−)-epicatechin, barbituric acid, and the cocrystal.

The Raman spectrum of cocrystal is compared with those of the free compounds to determine the bridging sites that may be involved in association. The hydroxyl groups are the possible bonding sites in EC with the carbonyl and amine groups of barbituric acid. Raman spectra of free EC are relatively complicated due to the interactions of the conjugated A/C ring system with benzene ring B and the presence of a H-bond network between the hydroxyl groups. These effects cause the widening and overlapping of many bands, which makes interpretation difficult. The Raman spectra of the EC-BTA cocrystal and the starting components are shown in Figure 7 and listed in Table S2 (Supporting Information). The spectral analysis was based on the assignments already reported for (−)-epicatechin, quercetin, and other flavonoids with a similar skeleton architecture.^37−42^ The Raman spectrum of the cocrystal is consistent with the presented structural data. The Raman spectrum of BTA showed two broad νN–H stretching vibrations with low intensity at 3195 and 3095 cm^–1^, whereas no absorption bands from νN–H and νO–H stretching vibrations were observed between 4000 and 3100 cm^–1^ in the Raman spectrum of EC-BTA. The fine structure spectrum in the cocrystal is displayed from 3100 to 2800 cm^–1^. The Raman scattering peaks centered at 3074, 3037, and 3018 cm^–1^ and 2932, 2916, and 2890 cm^–1^ belong to C–H stretching vibrations in phenyl and common C–H stretching in dihydropyran and the barbituric acid ring, respectively. The above bands remain unchanged compared to the spectra of free components. The main evidence for cocrystallization can be observed in the 1800–1700 cm^–1^ region related to the νC=O stretching mode. The peaks centered at 1734, 1719, and 1703 cm^–1^ are attributed to the C = O stretching vibrations in BTA. The highest frequency band is due to the νC_4,6_=O symmetric stretch, while the middle and the lowest are due to the νC_4,6_=O antisymmetric stretch and the νC_2_=O stretch, respectively.^43,44^ In the EC-BTA spectrum, these bands appear at 1742, 1717, and 1695 cm^–1^, respectively. Interestingly, the strongest peak at 1734 cm^–1^ shifts toward higher frequencies, whereas the remaining ones are slightly red-shifted. This suggests that BTA molecules are more strongly hydrogen-bonded in a free compound than in a cocrystal. The coupled δN–H bending and νC–N stretching vibrations at 1428 cm^–1^ shift to 1365 cm^–1^ in the cocrystal spectrum. The strong shift of this band toward lower frequencies is explained by the participation of the N–H group in an intermolecular hydrogen bond formed between the BTA carbonyl and OH groups in water molecules. Besides, new weak bands appear at 1809 and 1795 cm^–1^, which may indicate the presence of free C=O groups in the cocrystals. These spectral events prove that the BTA coformer participates in an association process with the (−)-epicatechin molecules. The C=O and N–H moieties are involved in the formation of hydrogen bonds between the (−)-epicatechin and coformer molecules.

Generally, the bands in the 1650–1500 cm^–1^ region of free EC are attributable to both aromatic rings, whereas most bands at lower Raman shifts can be mainly attributed to the A or B ring vibration mode (Table S2). A careful inspection of this region did not reveal any significant changes between the spectrum of the cocrystal and that of pure (−)-epicatechin. In the pharmaceutical term of cocrystals, the interaction between the API and the coformer showed only a hydrogen bond or a noncovalent bond that could be separated into the parent compounds during dissolution. Formation of a new compound during the cocrystallization process was not allowed; therefore, all components and the cocrystal should have the same spectral pattern in the 1500–500 cm^–1^ fingerprint region of the Raman spectrum. No obvious changes in the Raman spectra were detected in the specimens, and only a slight widening and an increase of the Raman scattering bands on inspection of this region in the EC-BTA spectrum were noticed (Figure 7).

### 2.5 Solubility Analysis

**Figure 8:**
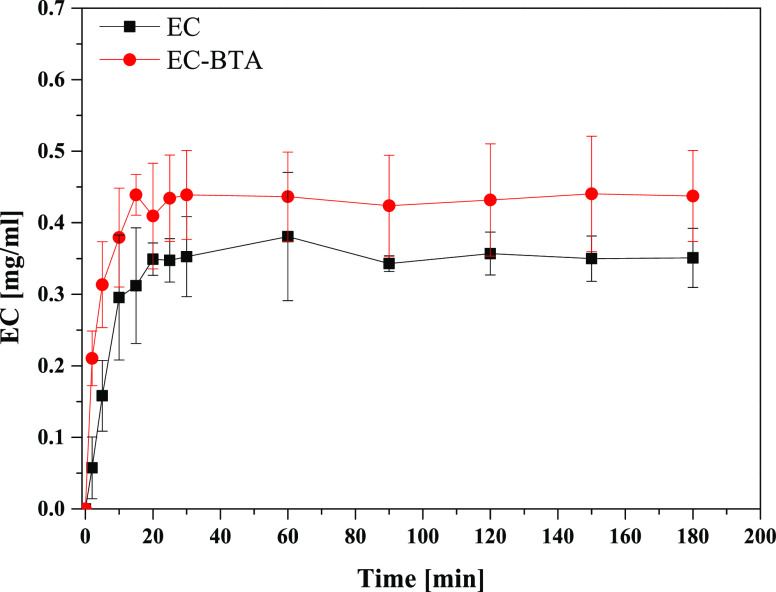
Solubility profiles of EC and its cocrystal EC-BTA in the PBS buffer solution. Error bars represent standard deviations obtained from triplicate measurements.

**Figure 9:**
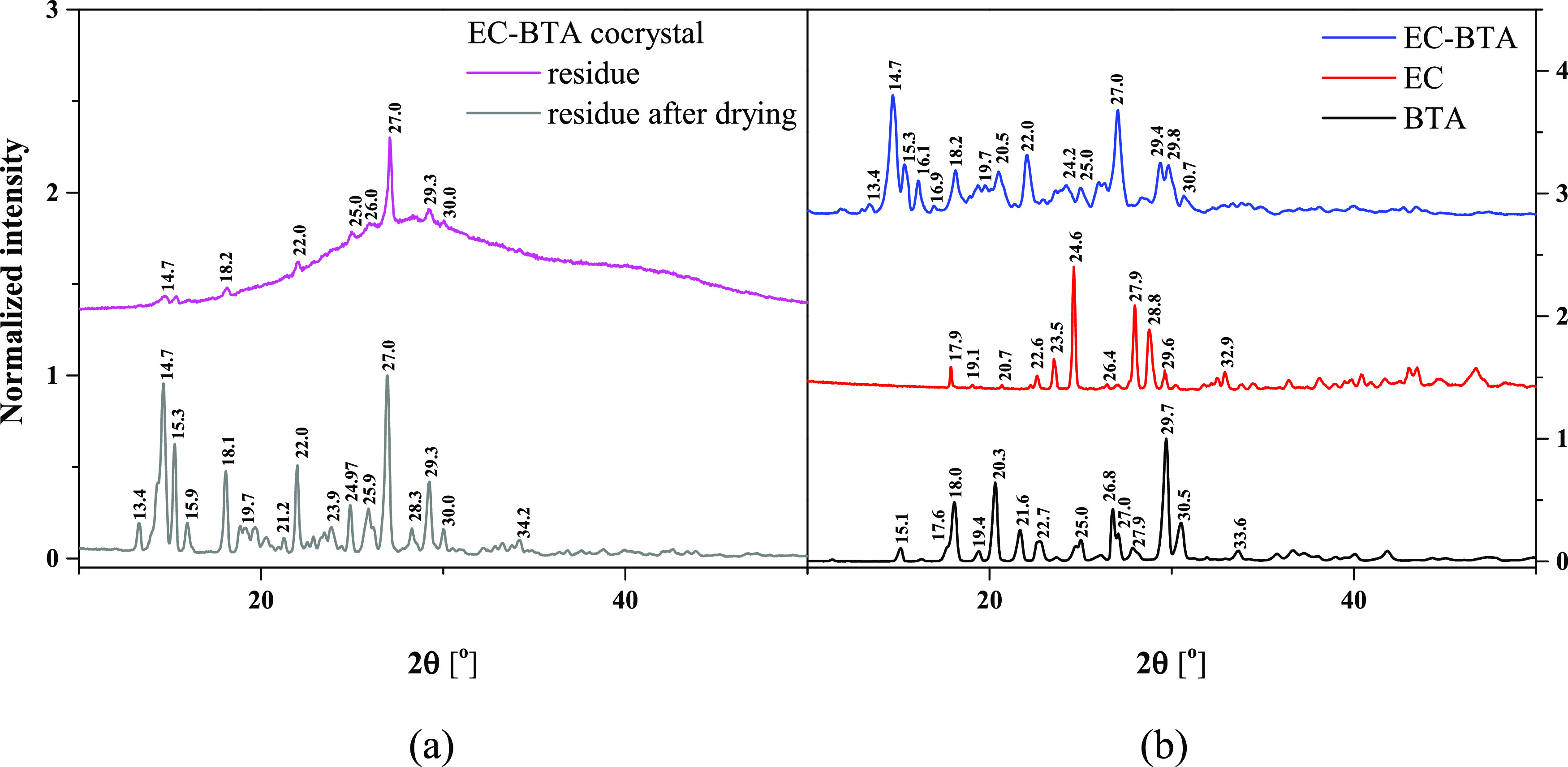
Normalized X-ray powder diffraction (PXRD) patterns of the EC-BTA cocrystal residue after dissolution in the buffer solution and after drying (panel a) and normalized X-ray powder diffraction (PXRD) patterns obtained for (−)-epicatechin, barbituric acid, and the EC-BTA cocrystal (panel b).

The results of the solubilities of (−)-epicatechin and its cocrystal in the buffer PBS medium (pH 7.4) are presented in Figure 8. The solubility curve reached a constant level in the PBS solution with the maximum solubility of 0.43 mg/mL based on pure (−)-epicatechin after 15 min for EC-BTA and 0.35 mg/mL after 20 min for EC. In this case, the solubilities for EC and EC-BTA were insignificantly close to each other. Although the solubility studies of pure EC and its cocrystal exhibited a 1.2-fold enhancement of the EC solubility in an aqueous solution for EC-BTA, the analysis of variance showed that there are no statistical differences (*p* > 0.05) between the dissociation rates. This may be due to a relatively large standard deviation. Moreover, the “spring and parachute” effect described by Guzmán et al.,^45^ which is commonly observed in the case of increased solubility of drug cocrystals, did not occur in the study described here. The PXRD analysis was performed on the residual material of the solubility analysis of EC-BTA after 1 h of the experiment to examine any significant changes in the phase transition of the cocrystal (Figure 9). The PXRD results showed that the wet residue (measurement immediately after removing the residue from the solution) is similar to the EC-BTA cocrystal. There were no other reflections that could indicate a change in the phase transition of the cocrystal. However, after drying the residues, the PXRD pattern showed a similarity to BTA and the EC-BTA cocrystal. Some cocrystal reflections are slightly shifted toward lower 2θ angles, which may indicate the formation of a new form of the EC-BTA cocrystal, probably the hydrate form.^46^

Furthermore, the influence of the environment pH on the solubility of (−)-epicatechin and its cocrystal EC-BTA was investigated. In Figure S3, the dissolution profile in the pH range 1–7 for EC and EC-BTA is presented. According to the published studies, catechins are generally stable under pH conditions ranging from 1.8 to 6.4 on the basis of the pH environment of the human gastrointestinal tract.^24,47^ Despite the fact that catechins are stable in acidic pH environments, in this paper the influence of cocrystallization on (−)-epicatechin solubility was studied. After 2 h of the experiment, comparable solubilities were obtained for both EC and EC-BTA in the tested pH range, which confirms the fact that both (−)-epicatechin and its cocrystal are stable in an acidic environment. In this case, the statistical analysis did not show any significant differences (*p* > 0.05) between EC and its cocrystal. It can be assumed that barbituric acid does not affect significantly the dissolution behavior of (−)-epicatechin in acidic and neutral media.

### 2.6 Hirshfeld Surface Analysis

**Figure 10:**
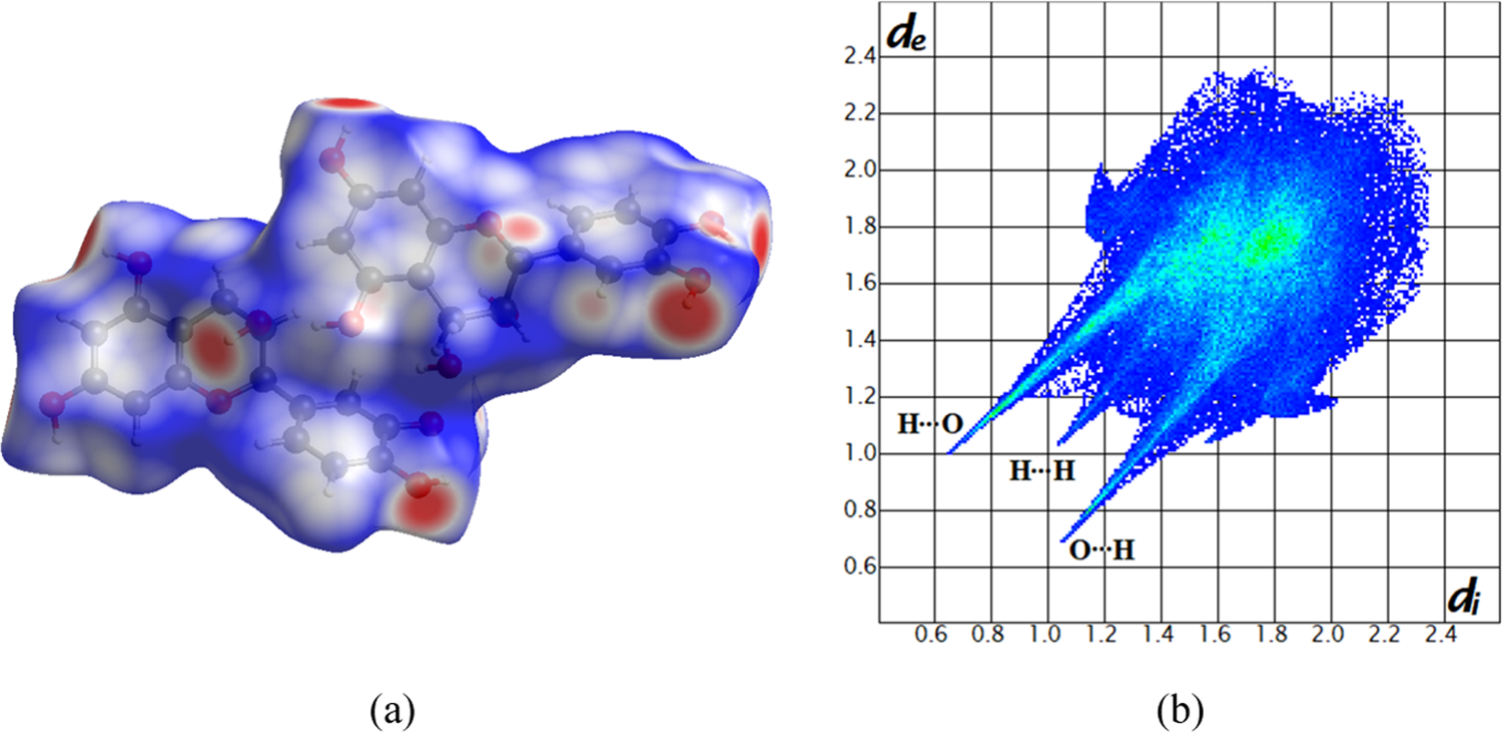
Views of *d*_norm_ (from 0.7 Å (blue) to −0.5 Å (red)) mapped on the Hirshfeld surface of (−)-epicatechin molecules (panel a) and the corresponding fingerprint plot (panel b).

**Figure 11:**
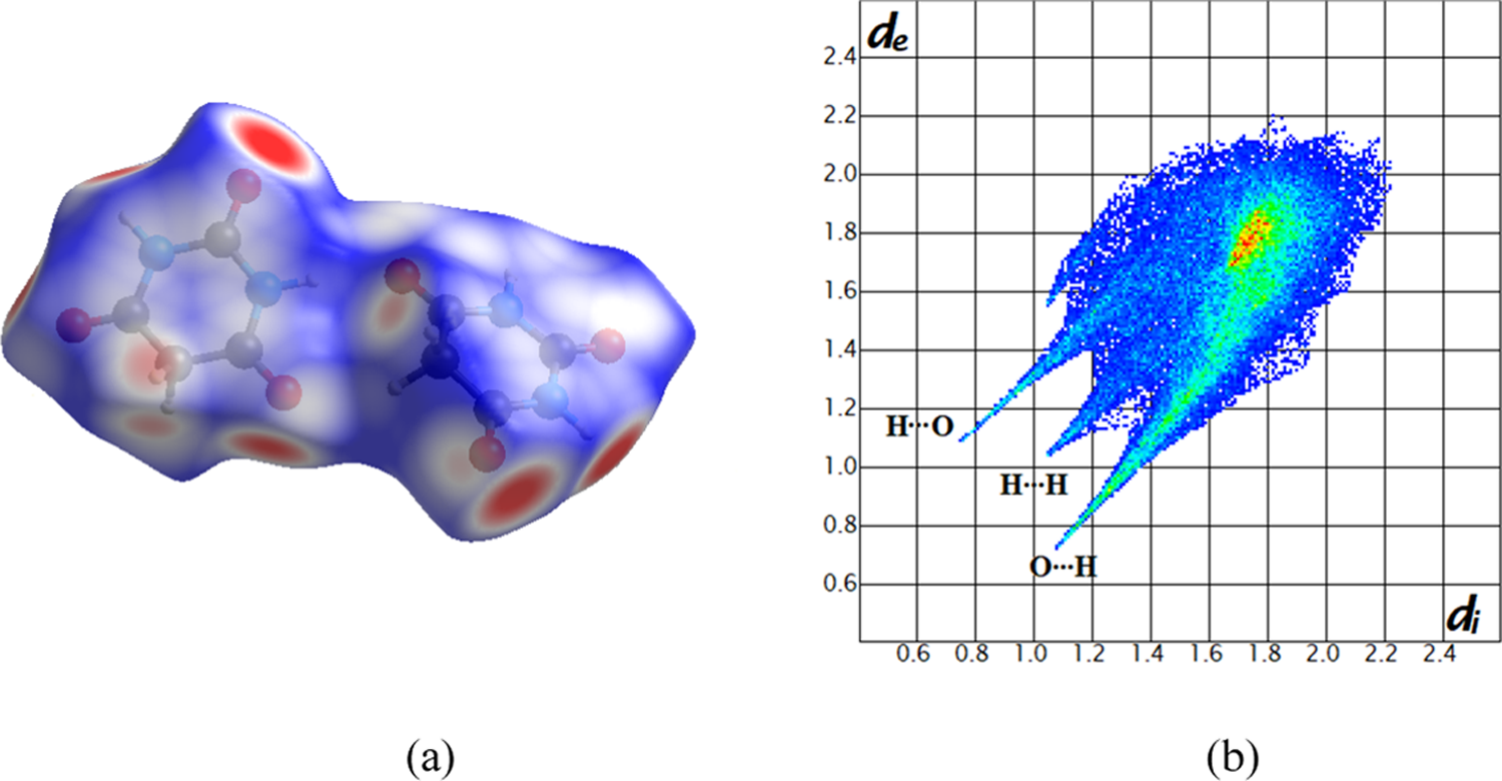
Views of *d*_norm_ (from 0.7 Å (blue) to −0.5 Å (red)) mapped on the Hirshfeld surface of barbituric acid molecules (panel a) and the corresponding fingerprint plot (panel b).

**Figure 12:**
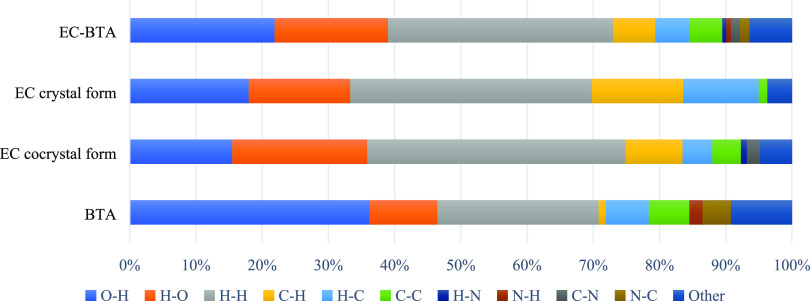
Relative contribution of chosen intermolecular interactions to the Hirshfeld surface area of EC-BTA and the EC crystal.

The Hirshfeld surfaces and molecular 2D fingerprints were calculated for the EC-BTA cocrystal and both pure components using CrystalExplorer v17.5. The Hirshfeld surface and 2D fingerprints for both independent molecules are presented in Figures 10 and 11. The results of the Hirshfeld surface analysis for the asymmetric unit cell of the EC-BTA cocrystal are presented in the Supporting Information (Figures S4 and S5, panels c and d). To understand better the effect of hydrogen bonding on the cocrystal solubility and to correlate with the experimental observations, the analysis of Hirshfeld surfaces was performed for the crystallographic data of the (−)-epicatechin crystal, which is available in the CCDC database (CIF number 1130393).^48^ The relative contributions of chosen intermolecular interactions to the Hirshfeld surface area of EC-BTA constituents, the EC-BTA cocrystal, and the (−)-epicatechin crystal are presented in Figure 12.

The analysis of (−)-epicatechin molecules’ interactions reveals that the greatest contribution to hydrogen bonding is provided by the intermolecular interactions O–H···O (35.9%), which can be observed on the Hirshfeld surface as red spots. The EC molecule is involved in the multiple hydrogen bonds comprising O–H···O homo- and heteromolecular interactions. A key role in the stabilization of the cocrystal is also played by the weak H···H interactions, whose contribution to all bonds on the surface is 39.0%. Some C···H contacts also have an influence on the total surface contribution, indicating the weak hydrogen interaction of C–H···π between molecules (13.1%). Moreover, the π···π stacking that constitutes 4.3% of the total interactions on the entire surface implies slight involvement of weak interactions between the EC molecule and the ring of barbituric acid. The contribution of other interactions is small, less than 2%. Strong hydrogen bonds O–H···O in the fingerprint diagram are marked as two spikes, which are of comparable length, indicating a similar donor/acceptor for these contacts (Figure 7b). The C···H/H···C shows a symmetric pair of wings, while the H···H contacts show asymmetric spots spread over a large area as broad peaks in the cocrystal.

The Hirshfeld surface analysis for the second component, barbituric acid, reveals that the major interactions are O···H/H···O, H···H, C···H/H···C, and C···C contacts with 46.5, 24.3, 7.6, and 6.1%, respectively. Acceptor (O–H···O) and donor (N–H···O) interactions (see Table 3) can be seen on the Hirshfeld surfaces as red areas.

**Table 3:** Specification of Intermolecular Hydrogen Bonds Observed in the EC-BTA Cocrystal

D–H···A	D–H (Å)	H···A (Å)	angle (deg)	symmetry
O6B–H12B···O4B	0.819	2.020	147.14	*x*, −1 + *y*, 1 + *z*
O6A–H12A···O4A	0.822	2.067	145.66	*x*, −1 + *y*, 1 + *z*
O3A–H6A···O2B	0.821	1.935	171.49	*x*, *y*, *z*
O3B–H6B···O2A	0.949	1.784	175.03	*x*, 1 + *y*, −1 + *z*
O4B–H8B···O1C	0.822	1.955	156.51	2 + *x*, *y*, 1 + *z*
O4A–H8A···O3C	0.796	2.030	155.80	*x*, *y*, *z*
O3D···H2C–N2C	0,860	2.067	164.00	*x*, *y*, *z*
N1D–H1D···O2C	0.860	2.167	158.46	–1 + *x*, *y*, *z*
π···π	distance in face-to-face stacking			
C5A···C3C	3.387			*x*, −1 + *y*, *z*
C4A···C2C	3.384			*x*, −1 + *y*, *z*
C7A···C1C	3.385			*x*, −1 + *y*, *z*
C2D···C11A	3.362			*x*, −1 + *y*, *z*

Hydrogen bonding is one of the vital factors that can affect the stabilization of the crystalline structure and determine the solubility properties of the cocrystal.^49^ The reduced quantity of intermolecular hydrogen bonding in the cocrystal structure in comparison to pure API can weaken significantly the crystal packing and increase the solubility.^50,51^ The hydrogen bonding effect on the dissolution profile is governed by multiple factors that can increase and decrease the drug kinetic solubility. For greater insight into the structure–solubility relation between the EC-BTA cocrystal and the (−)-epicatechin crystal, the analysis of intermolecular interactions is required. It is worth noting that the overall contribution of the O···H/H···O interactions in the (−)-epicatechin crystal is 33.3%, which is slightly lower than those in the EC cocrystal component (35.9%) and the EC-BTA cocrystal (39.0%). On the other hand, the contribution of nonpolar interaction (C···C, C···H/H···C) for the (−)-epicatechin crystal is much higher than that in EC-BTA (26.5 and 16.5%). Even though the contribution of hydrogen bonding in the (−)-epicatechin crystal is slightly lower in comparison to that in the cocrystal, the presence of a greater number of nonpolar interactions may result in a slightly lower solubility concerning the cocrystal. Comparing the data obtained from the dissolution analysis combined with the statistical test and the Hirshfeld surface analysis, it can be assumed that the solubilities of (−)-epicatechin and the EC-BTA cocrystal are comparable.

## 3 Conclusions

In summary, a novel cocrystal of (−)-epicatechin with barbituric acid in a 1:1 stoichiometry was obtained by the slow solvent-evaporation technique. The chemical characteristics of the cocrystal were confirmed by single-crystal X-ray diffraction, PXRD, TGA–DSC, and Raman spectroscopy. EC-BTA crystallized in the space group *P*1 with two molecules each of EC and BTA and three water molecules in the asymmetric unit. The analysis of packing and interactions in the crystal lattice revealed that molecules in the target cocrystal were packed into tapes formed by the O–H···O type contact between the (−)-epicatechin and coformer molecules. The Raman spectra confirm that the C=O and N–H moieties of barbituric acid are engaged in the formation of hydrogen bonds between the (−)-epicatechin and coformer molecules. In addition, the solubility studies of pure EC and the EC-BTA cocrystal exhibited minor enhancement of the EC solubility in the buffer PBS and in the pH range of 1–7. It can be assumed that pure (−)-epicatechin and its cocrystal have stable levels of solubility in an acidic environment. The statistical analysis showed no significant differences in the solubility profiles in the PBS buffer and different pH media in the range of 1–7 between (−)-epicatechin and the EC-BTA cocrystal. In this case, the cocrystallization method does not contribute to considerable improvement of the EC solubility. Nevertheless, it can be assumed that the lipophilic coformer, which is barbituric acid, can enhance the membrane permeability.^52,53^ Moreover, the determination of the permeability through biological membranes by the new molecular form of EC in comparison with those of pure compounds will be the subject of the next paper.

## 4 Experimental Section

### 4.1 Materials

(−)-Epicatechin of ≥90% and (+)-catechin hydrate of ≥96% purities were purchased from Sigma-Aldrich (Saint Louis, MO) and recrystallized from ethanol. Barbituric acid, 5-methylo-1*H*-benzotriazole, 4-(methyloamino)-pyridine, 5-methylbenzimidazole, acetamide, salicylamide, niacinamide, isonicotinamide, caffeine, glutarimide, and the solvents purchased from Sigma-Aldrich were of analytical grade.

The cocrystal synthesis: Catechins and the BTA coformer in 1:1 and 2:1 molar ratios were dissolved in ethanol at 30 °C. Colorless crystals were obtained by slow evaporation of the solvent. The procedure lasted for 2 days at room temperature. Crystals were collected from the crystallization vessels.

### 4.2 Methods

#### 4.2.1 Single-Crystal X-ray Diffraction

Single-crystal X-ray diffraction data were collected on a Rigaku Oxford Diffraction diffractometer equipped with a MicroMax-007 HF, with a twisted Cu anode as an X-ray source (Cu Kα), multilayer optics, and a Pilatus 300 K surface detector at *T* = 293 K. 2θ was measured in the range of 6–110° with a resolution of 0.078° and 10 min count time per frame. Data reduction and cell refinement were performed with CRYSALIS^PRO^.^54^ All structures were solved with direct methods^55^ and refined using the Olex2 software.^56^ The refinement was based on the square structure factors (*F*^2^) for all reflections except those with very negative *F*^2^ values. Almost all of the hydrogen atoms were in an idealized geometric position except for those forming the hydrogen bonds. Table 1 lists the experimental details for all measured single crystals. The crystallographic data were deposited at the Cambridge Crystallographic Data Center (CCDC) at No. 2047274.

#### 4.2.2 X-ray Powder Diffraction (PXRD)

The PXRD patterns of (−)-epicatechin, the coformer, and the cocrystal were obtained using the same diffractometer as that used for the single-crystal analysis (Rigaku, Tokyo, Japan) but working in a powder diffraction mode. The measured range was 10–90°. For averaging, the sample was rotated around the phi axis. The data were collected using the CRYSALIS^PRO^ software.^54^

#### 4.2.3 Determination of Melting Points

Melting points of the compounds were estimated using the Büchi melting point B-540 apparatus. A small amount of sample was used to fill a capillary tube. The starting temperature was set at 200 °C, and the heating rate was 5 °C/min.

#### 4.2.4 TGA–DSC Thermal Analysis

Thermogravimetric and differential scanning calorimetry measurements (TGA–DSC) were performed using a Setaram SETSYS 16/18 analyzer. The 3–5 mg samples were heated in aluminum sample pans in a dynamic air atmosphere (*v* = 0.75 L/h). The temperature range was set at 10–700 °C with a heating rate of 10 °C/min.

#### 4.2.5 Raman Spectroscopy

Raman spectra of solid samples were recorded using a Nicolet 8700 FT-IR/NXR FT-Raman system (Thermo Scientific) in the range of 100–4000 cm^–1^, using a Micro Stage attachment, a 1064 nm diode laser, and an InGaAs detector. The resident Omnic software was used to collect and process Raman spectra.

#### 4.2.6 Hirshfeld Surface Analysis

The Hirshfeld surface analysis and the 2D fingerprint plot were generated using a CrystalExplorer 17.5 tool. The graphs were used to describe various intermolecular interactions, especially H···H bonds, which are most important in the stabilization of the crystal lattice and other interactions occurring in the EC-BTA molecule. A crystallographic data (CIF) file was used as input data for the analysis. The directions and forces of the intermolecular interactions in the crystals were mapped on the Hirshfeld surfaces as described by Abidi et al.^57^

#### 4.2.7 Dissolution Analysis

The solubilities of pure (−)-epicatechin and its cocrystal were determined according to the method used by Shimpi et al.^58^ with slight modification of the amount of ingredients. Briefly, 4 mg of EC powder and the cocrystal were suspended in 10 mL of PBS buffer (pH 7.4). The samples were mixed in a thermostatic vessel at 25 °C and with 100 rpm orbital shaking. Aliquots of the samples were transferred from the suspension at intervals of 5, 10, 15, 20, 25, 30, 60, 90, 120, 150, and 180 min and then filtered through a 0.22 μm PTFE filter. Electronic absorption spectra were recorded using a Cary 300 Bio (Varian) UV–Vis Cary 300 Bio double-beam spectrophotometer equipped with a Cary Peltier temperature controller. The samples were measured in closed quartz (Helma) cuvettes with a path length of 1.0 cm in the wavelength range of 200–600 nm. The absorption coefficient for EC (ε = 3993.21 dm^3^/mol cm) was determined from the slope of the absorbance measured at 278 nm as a function of the EC concentration in the PBS buffer. After the solubility analysis, the solid EC-BTA residues were collected at room temperature for the analysis using powder X-ray diffraction (PXRD).

##### 4.2.7.1 pH Measurements

The solubilities of the EC-BTA cocrystal and EC were measured in a pH range of 1–7, and the pH of the solution was adjusted using 1 M HCl or 1 M NaOH. Briefly, 1 mg of EC powder and the cocrystal were suspended in 5 mL of water solution. The samples were mixed in a thermostatic vessel at 25 °C and with 100 rpm orbital shaking. After 2 h, the sample solution was filtered (using 0.22 μm PTFE filter) and analyzed using the UV–vis spectrophotometer.

#### 4.2.8 Statistical Analysis

All data obtained from the solubility analysis were analyzed using the Statistica 13 software (TIBCO Software Inc. Palo Alto, CA). To find out whether the different results between EC and EC-BTA are statistically significant, a two-way variance analysis (ANOVA) test was performed. In the results, the significant level was assumed at α = 0.05.

## Supporting Information Available

Raman data (band assignments) for individual components and target cocrystals and Hirshfeld surface analysis; and DCS plots and PXRD patterns (PDF)
CCDC code 2047274 (CIF)

## Supplementary Material

ao0c06239_si_001.pdf

ao0c06239_si_002.cif
