# Supplementary material for: Synthesis and Characterization of a (−)-Epicatechin and Barbituric Acid Cocrystal: Single-Crystal X-ray Diffraction and Vibrational Spectroscopic Studies
Source: ACS Omega. 2021 Mar 16;6(12):8199–209. doi: 10.1021/acsomega.0c06239 (PMC8014927; doi:10.1021/acsomega.0c06239)
Supplement: Supplementary file 1 — ao0c06239_si_001.pdf [file ao0c06239_si_001.pdf]

Supporting Information

Synthesis and characterization of a (-)-epicatechin and barbituric acid cocrystal: Single crystal X-Ray diffraction and vibrational spectroscopic study

Iwona Budziak-Wieczorek[a*], Urszula Maciołek[b]

[a] Department of Chemistry, University of Life Sciences in Lublin, Akademicka 15, 20-950 Lublin, Poland

[b] Analytical Laboratory, Institute of Chemical Sciences, Faculty of Chemistry, Maria Curie-Sklodowska University, pl. M. Curie-Skłodowskiej 3, 20-031 Lublin, Poland

* Correspondence author: iwona.budziak@up.lublin.pl

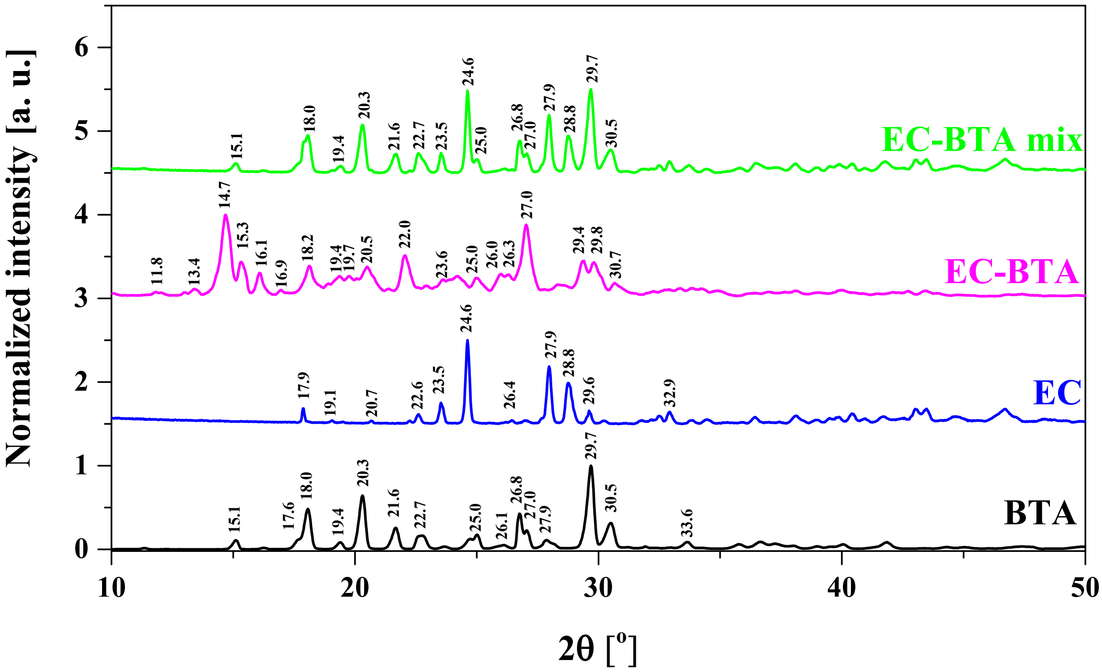

Figure S1. Normalized X-ray powder diffraction patterns obtained for (-)-epicatechin, barbituric acid, EC-BTA cocrystal and physical mixture of EC and BTA.

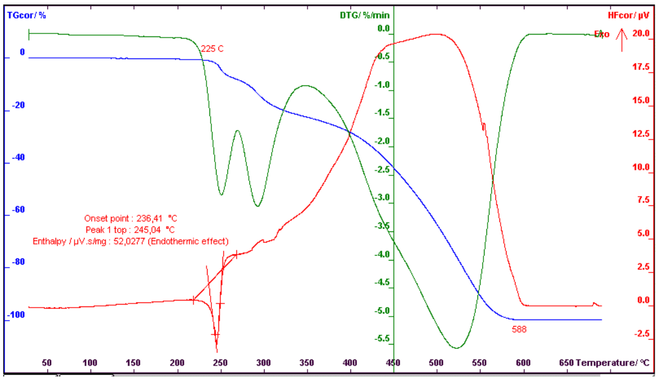

(a)

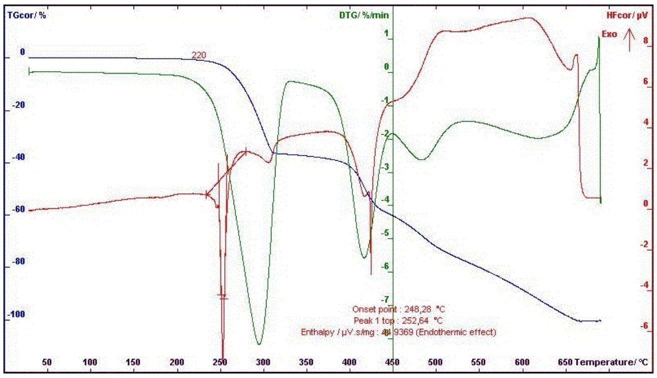

(b)

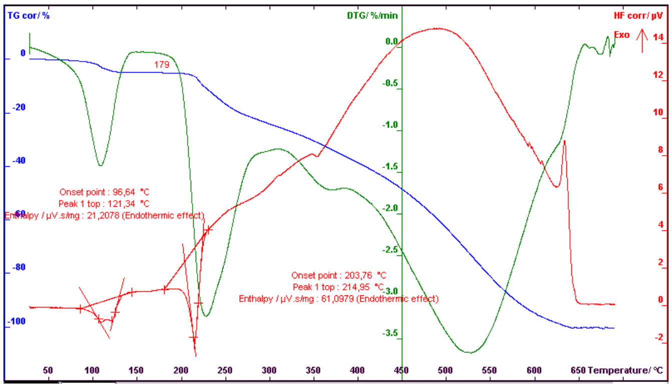

(c)

Figure S2. Thermogravimetric analysis (TGA) and differential scanning calorimetry thermograms of (-)-epicatechin (panel a), barbituric acid (panel b), EC-BTA cocrystal (panel c) recorded at scan rates of 10°C/min.

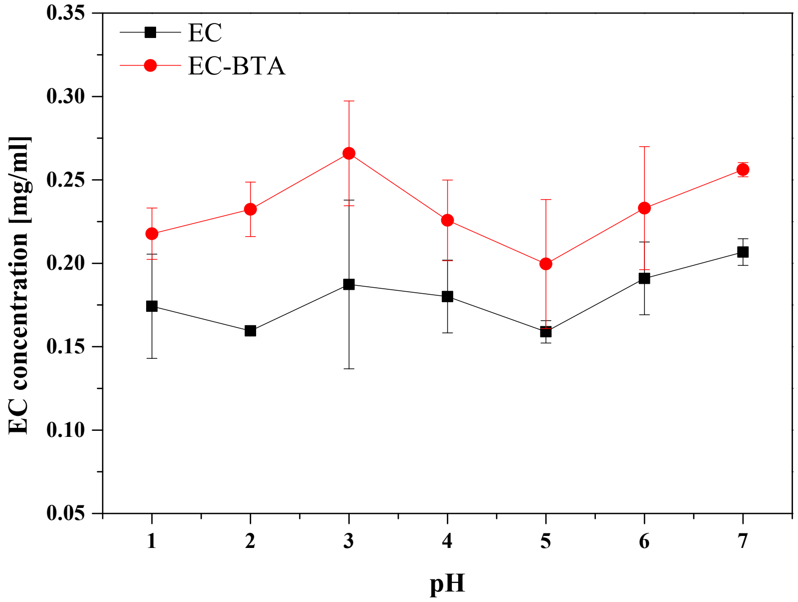

Figure S3. Solubility-pH dependence of 1:1 EC-BTA cocrystal and EC in the pH range 1-7. Error bars represent standard deviations obtained from triplicate measurements.

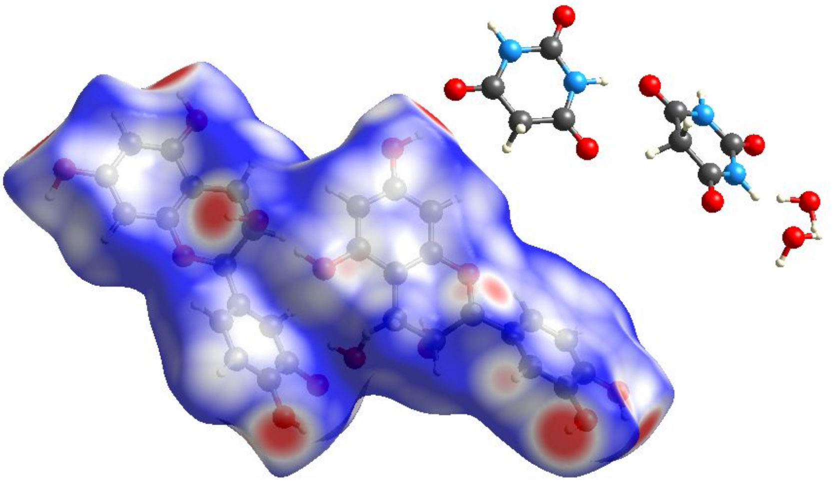

Figure S4. Views of d_{norm} (from 0.7 Å (blue) to -0.5 A (red)), mapped on the Hirshfeld surface for the asymmetric unit cell of the EC-BTA cocrystals.

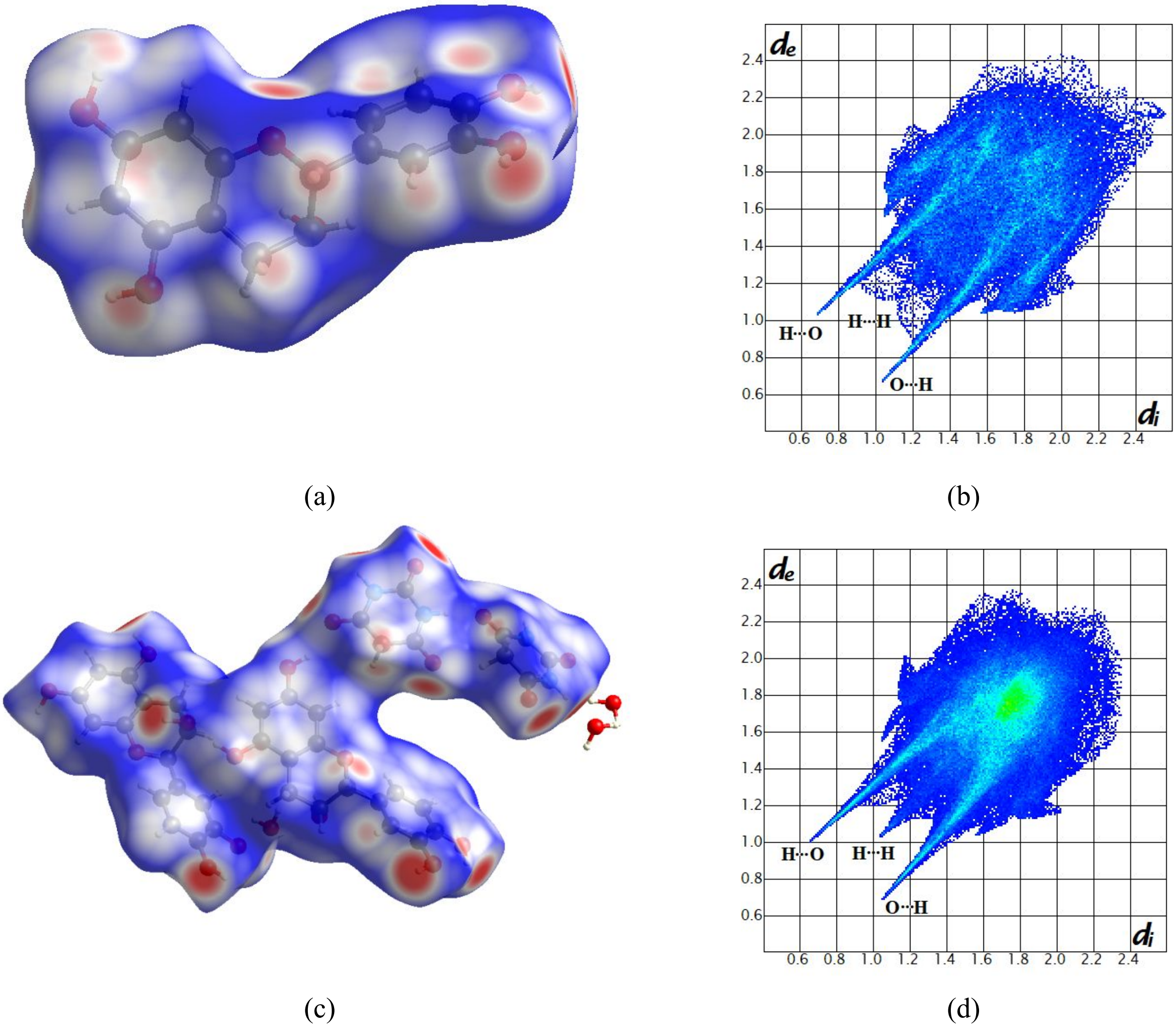

Figure S5. Views of d_{norm} (from 0.7 Å (blue) to -0.5 A (red)), mapped on the Hirshfeld surface and corresponding fingerprint plot for the (-)-epicatechin crystal (panels a and b), EC-BTA cocrystal (panels c and d).

Table S1. Bond length for EC-BTA.

Atoms	Length/Å	Atoms	Length/Å
O1A-C1A	1.438(6)	O1B-C1B	1.437(6)
O1A-C9A	1.375(6)	O1B-C9B	1.364(6)
O2A-C2A	1.412(7)	O2B-C2B	1.425(6)
O3A-C5A	1.358(6)	O3B-C5B	1.367(6)
O4A-C7A	1.362(6)	O4B-C7B	1.371(6)
O5A-C12A	1.357(7)	O5B-C12B	1.345(7)
O6A-C13A	1.372(6)	O6B-C13B	1.365(6)
C1A-C2A	1.520(8)	C1B-C2B	1.511(8)
C2A-C3A	1.528(6)	C2B-C3B	1.526(7)
C3A-C4A	1.492(7)	C3B-C4B	1.501(7)
C4A-C5A	1.411(7)	C4B-C5B	1.407(7)
C5A-C6A	1.396(7)	C5B-C6B	1.385(7)

C6A-C7A	1.382(7)	C6B-C7B	1.385(7)
C7A-C8A	1.385(7)	C7B-C8B	1.393(7)
C8A-C9A	1.388(7)	C8B-C9B	1.389(7)
C4A-C9A	1.393(7)	C4B-C9B	1.385(7)
C1A-C10A	1.516(6)	C1B-C10B	1.497(7)
C10A-C11A	1.379(7)	C10B-C11B	1.392(7)
C11A-C12A	1.383(7)	C11B-C12B	1.395(7)
C12A-C13A	1.409(7)	C12B-C13B	1.392(8)
C13A-C14A	1.366(8)	C13B-C14B	1.375(7)
C14A-C15A	1.380(7)	C14B-C15B	1.384(7)
C15A-C10A	1.390(7)	C15B-C10B	1.396(7)
O1C-C1C	1.219(8)	O1D-C1D	1.217(7)
O3C-C4C	1.222(8)	O3D-C4D	1.216(8)
O2C-C2C	1.224(7)	O2D-C2D	1.213(9)
N2C-C1C	1.373(8)	N2D-C1D	1.407(8)
N2C-C2C	1.357(7)	N2D-C2D	1.380(8)
N1C-C1C	1.389(8)	N1D-C1D	1.385(8)
N1C-C4C	1.387(8)	N1D-C4D	1.350(8)
C3C-C2C	1.461(8)	C3D-C2D	1.461(10)
C3C-C4C	1.435(8)	C3D-C4D	1.428(10)

Table S2. Raman bands (cm^{-1}) observed in the FT Raman spectra of BTA, EC and EC-BTA[a] (solids) with spectral band assignments.

Assigments	EC-BTA	EC[a]	BTA[b]
ν_{ring}BTA, ν(N–H)	-	-	3195 w, br
ν_{ring}BTA, ν(N–H)	-	-	3095 w, br
ν_{ring} (A and B), ν(=C–H)	3074 m	3073 m	-
ν_{ring}(A and B), ν(=C–H)	3037 w	3038 w	-
ν_{ring}(A and B), ν(=C–H)	3018 w	3018 w	-
ν_{ring}BTA, ν_{comb}(N-H)	-	-	2997 w
ν_{ring}C, $\nu_a(CH_2)$	-	2946 vs	-
ν_{ring}C, ν(C–H)	2932 vs	2932 vs	-
ν_{ring}BTA, $\nu_a(CH_2)$	2916 vs	2917 s	2912 w
ν_{ring}(BTA and C), $\nu_s(CH_2)$	2880 m	2880 w	2878 w
ν_{ring}BTA, $\nu_a(C_{4,6}{=}O)$	1742 s	-	1734 s
ν_{ring}BTA, $\nu_s(C_{4,6}{=}O)$	1717 m	-	1719 m
ν_{ring}BTA, $\nu(C_2{=}O)$	1692 m	-	1703 m
ν_{ring} (A and B)	1616 vs	1616 vs	-
ν_{ring} (A and B)	1519 vw	1520 vw	-
ν_{ring} A, ν(C–OH)	1434 vw	1434 vw	-
ν_{ring}BTA, ν(C–N)	-	-	1428 w, br
ν_{ring}BTA, $\nu(CH_2$ wagging)	-	-	1390 w
ν_{ring}B, ν(C–C), δ(C–OH);<br>ν_{ring} BTA, ν(N–H)	1362 m	1365 w	1370 vw
ν_{ring} A, δ(C–OH); δ_{ring} (N–H)	1343 m	1341 m	1347 vw
ν_{ring} A; ν(C–O, C–C);	1285 m	1284 m	1285 w

ν_{ring}BTA, ν (C–N,)			
ν_{ring} A; Δ(C–C–C); ν(C–O); ν_{ring}BTA, ν(C–N)	1179 m	1178 m	1176 w
Δ(C–C–C); ν(C–O)	1094 m	1093 w	-
Δ(C–C–C); ν(C–O)	1068 w	1069 w	-
ν_{ring} C; ν(C–O–C)	1016 s	1016 s	-
ν_{ring} (A and B)	982 w	979 w	-
ν_{ring} (A and B)	964 w	963 w	-
ν_{ring}BTA, ν(C–C)	-	-	932 w, br
ν_{ring} (A, B, C), ν(C–C)	901 vw, br	902 vw	-
ν_{ring} (A, B, C), ν(C–C)	841 w, br	841 w	-
γ(=C–H), Γ_{ring} B	794 s, sh	794 m, sh	-
Δ(C–O–C), Δ(C–C–C), γ(=C–H), Γ_{ring} B	780 vs	779 vs	-
Δ(C–C–C), γ(=C–H)	723 s	723 s	-
ν_{ring} BTA breathing, γ(O–H)	664 vs, br	672 m	664 vs
Δ(C–C–C), γ(O–H)	648 m, sh	648 m	-
Δ(A,C), γ(N-H), γ(C=O)	598 m, br	596 m, br	617 w,br
Δ(A, B, C)	548 vw,br	548 w,br	-
Δ_{ring} BTA bending	507	-	508

Abbreviations: *s* = strong; vs = very strong; *m* = medium; *w* =weak; *vw* =very weak; *sh* = shoulder; br = broad; ν_a –asymeric stretching; ν_s –symeric stretching; ν_a –combination stretching; *δ* – in plane bending; *γ* – out of plane bending; *Δ* – skeleton in plane bending; *Γ* – skeleton out of plane bending; A and B are related to benzene rings; C– pyrone ring.
